# HISTÓRIA, CIÊNCIAS, SAÚDE – MANGUINHOS EM 2022

**DOI:** 10.1590/S0104-59702023000100026

**Published:** 2023-07-10

**Authors:** Marcos Cueto

**Affiliations:** i Casa de Oswaldo Cruz Fiocruz Rio de Janeiro RJ Brasil Editor científico, investigador, Casa de Oswaldo Cruz/Fiocruz. Rio de Janeiro – RJ – Brasil

Como lo hemos hecho en los últimos años, quiero presentar un resumen de las actividades de la revista en el 2022 ( Cueto, 2021 , 2022 ). A pesar del contexto político de ese año, marcado por un gobierno negacionista que complicaba la difusión de la investigación científica, conseguimos mantener la regularidad y la calidad de los artículos del volumen 29 de nuestra revista.

Tuvimos varios textos y un conjunto de artículos fundamentales. Quisiera resaltar algunos, sin desmedro de otros excelentes.

El suplemento “Reciprocidades em desequilíbrio: história das relações entre animais”, publicado en el volumen 28, tuvo, a lo largo del 2022, una buena repercusión, con un elevado número de accesos a sus textos, lo que sugiere un interés especial de los lectores de la revista por este tema, de ahí que se destaque de nuevo en el editorial. Sospecho que su amplia repercusión se debe al creciente interés holístico sobre el futuro de la vida en la tierra que ha llevado a enarbolar propuestas como One Health y la salud planetaria ( Biehl, 2021 ), que entrelazan en un solo esfuerzo la comprensión y el cuidado del medio ambiente, de la naturaleza y de los seres humanos, además de los crecientes debates en la historiografía sobre el tema. Sus editores fueron Regina Horta Duarte, profesora de la Universidade Federal de Minas Gerais, Gabriel Lopes, de la Casa de Oswaldo Cruz, Natascha Stefania Carvalho De Ostos, del Instituto René Rachou/Fiocruz Minas, y Nelson Aprobato Filho, de la Universidade de São Paulo.

No menos importante fue la primera carta de editor del volumen 29 de comienzos de 2022, que reprodujo un editorial colectivo del fórum de editores de revistas académicas de la Associação Nacional de História (Anpuh) titulado “Por uma política de valorização das revistas acadêmicas na área de História”. Estaba dirigido a resaltar el papel de las revistas científicas de historia en la circulación del conocimiento, a reivindicar la validez de las evidencias recogidas en archivos y la legitimidad de las interpretaciones de los historiadores, y a llamar la atención sobre la importancia de sustentar los equipos editoriales que elaboran las publicaciones periódicas (no solo editores sino revisores, traductores, secretarios, programadores visuales, periodistas, entre otros).

De gran impacto también fue el dossier “Cultura material de la ciencia iberoamericana: museos, jardines y gabinetes científicos”, organizado por las investigadoras Carolina Valenzuela, de la Universidad de Chile, y María Gabriela Mayoni, de la Universidad de Buenos Aires, que apareció en el número 3 del año pasado. Los valiosos textos publicados son versiones mejoradas presentadas en el simposio Visual, Material and Sensory Cultures of Science, que fue parte de la importante novena conferencia de la Sociedad Europea de Historia de la Ciencia, realizada entre fines de agosto y comienzos de septiembre del 2020 en Bolonia, Italia. Al publicarlo queríamos reiterar, además, el compromiso de la revista con el registro de lo que viene ocurriendo en las fronteras del conocimiento que se hace público generalmente en eventos académicos.

Antes de ser llamada a ocupar el cargo de ministra de Salud, tuvimos el privilegio de publicar, en el número 4, una carta de Nisia Trindade Lima en la que celebraba los 21 años del Programa de Posgrado en Historia de las Ciencias y de la Salud de la Casa de Oswaldo Cruz. En su texto, argumenta que la investigación histórica es como una ventana para vislumbrar el futuro.

Finalmente, quisiera resaltar el número especial “Histórias transculturais de psicoterapias: novas narrativas”, organizado por los profesores Sonu Shamdasani y Cristiana Facchinetti. Este suplemento del 2022 es importante por dos motivos. Primero porque presenta las complejidades de la circulación e intercambio de conocimientos y prácticas de salud mental en Argentina, Brasil, los EEUU, Italia, Japón y Suiza. Segundo, porque es la expresión de un productivo acuerdo de colaboración que se remonta a 2016 entre University College London, donde es profesor Sonu Shamdasani, y la Casa Oswaldo Cruz, donde es investigadora Cristiana Facchinetti.

Todo lo anterior ha sido posible gracias al equipo editorial que regresó a trabajar al campus de Manguinhos de una manera presencial o semipresencial (Mônica Auler, Roberta Cerqueira, Camilo Papi, Mônica Cruz Caminha, Vinícius Renaud, Marciel Mendonça, Miriam Junghans y Fernando Vasconcelos). Ellos, y quien firma, interactuamos con las periodistas científicas Marina Leme y Vivian Mannheimer, que también son parte de nuestro equipo. Su trabajo fue fundamental para la repercusión de los textos de la revista porque difundieron el lanzamiento de la revista, alimentaron el blog que se está convirtiendo en una referencia para otros investigadores que lo citan, publicaron noticias de textos con “ganchos” específicos que atraen la mirada de los lectores y vincularon efemérides y personajes conocidos a artículos de la revista. Gracias a su labor, y por su puesto a los lectores, el blog nacional de la revista tuvo más de 460 mil visitas durante el 2022 (en el 2021 tuvo 389.797). Y hasta mayo de 2023, contábamos con más de 10.500 seguidores en la página de Facebook nacional (publicada en portugués). Por su lado, la cuenta internacional de Facebook (con noticias en español e inglés) tiene actualmente 5.200 seguidores, un aumento significativo frente a los 4.900 del 2021. En Twitter – donde publicamos contenido en portugués, inglés y español – actualmente contamos con 4.255 seguidores, un incremento de casi 500 seguidores respecto a inicios del 2022. Una característica importante de este medio de comunicación social es que a diferencia de años anteriores en los que la audiencia de la revista se formaba básicamente por lectores de países latinoamericanos, ahora la mayoría (30%) se concentra en EEUU, seguido de Brasil (15%) y el resto del porcentaje se lo reparten lectores de México, Argentina, Perú, Portugal, España y otros países. De esta manera, el blog contribuye de una manera decisiva a la internacionalización de la revista; un logro importante conseguido después de 10 años de su creación.

Esta internacionalización se está comenzando a sentir más lentamente en las sumisiones. En el 2022 fueron sometidos y procesados 172 textos para todas las secciones de la revista, de los cuales 67% procedían de autores y autoras de Brasil. Los países que seguían en la lista de mayor número de sumisiones fueron Portugal, Argentina, Colombia y Chile. Asimismo, es de destacar que, en el 2022, EEUU, Reino Unido, España, Uruguay y Perú sometieron, cada uno, entre dos y cinco artículos. En términos globales, lo anterior no significó una disminución con respecto al 2021, porque en ese año fueron recibidos 165 manuscritos.

Acabo escribiendo algunas frases pensando en el futuro inmediato. En los primeros meses del 2023 implementamos la publicación por flujo continuo. Este sistema consiste en la publicación electrónica de los artículos de la revista, individualmente o en pequeños grupos, a medida que se aprueban y procesan internamente (es decir, luego de una cuidadosa revisión lingüística, normalización de las referencias, tablas e imágenes, y otras tareas de los correctores). Ello viene a reemplazar la publicación de los artículos por número valiendo de ahora adelante solamente el volumen que indica el año. Las ventajas del flujo continuo son que permiten la difusión de la investigación de nuestros autores más rápidamente y aumentan la visibilidad de la revista. Asimismo, avizoramos renovados compromisos con la ciencia abierta, nuevas instrucciones a los autores y el fin de la sección “Testimonios de Covid-19” – lo que no significa que dejemos de recibir trabajos sobre el tema para todas las secciones. Es bueno recalcar que coincidimos con las recomendaciones de las autoridades sanitarias, especialmente en relación a la vacunación, quienes advierten que no sería sorprendente un recrudecimiento de una dolencia que todavía existe.

Estamos muy agradecidos a todos los autores, evaluadores, editores de secciones y miembros del cuerpo editorial que permitieron que nuestra publicación periódica tenga una visibilidad y un prestigio especial en el Brasil y buena parte del mundo. Finalmente, nuestro reconocimiento especial a Marcos José de Araújo Pinheiro, director da Casa de Oswaldo Cruz, la unidad de Fiocruz donde trabajamos, por el gran apoyo a la revista.
