# Supplementary material for: HISTÓRIA, CIÊNCIAS, SAÚDE – MANGUINHOS EM 2022
Source: Hist Cienc Saude Manguinhos. 2023 Jul 10;30:e2023026. [Article in Spanish] doi: 10.1590/S0104-59702023000100026 (PMC10395597; doi:10.1590/S0104-59702023000100026)
Supplement: Translation

# Translation: HISTÓRIA, CIÊNCIAS, SAÚDE – MANGUINHOS IN 2022

**DOI:** 10.1590/S0104-59702023000100026en

**Authors:** Marcos Cueto

**Affiliations:** i Brasil Scientific editor, researcher, Casa de Oswaldo Cruz/Fiocruz Rio de Janeiro – RJ – Brasil

As we have done in recent years, I would like to submit a summary of the journal’s activities in 2022 ( Cueto, 2021 , 2022 ). Despite the political context of that year, marked by a denialist administration that complicated the dissemination of scientific research, we managed to maintain the regularity and quality of the articles in volume 29 of our journal.

We had several texts and a set of fundamental papers. I would like to highlight some of them, without detriment from other excellent ones.

The supplement “Unbalanced reciprocities: the history of relationships between animals” published in our volume 28, had throughout 2022 a good repercussion, with a high number of accesses to its texts, suggesting a special interest of the journal’s readers, hence it stands out again in the editorial. I have a feeling that its wide repercussion is due to the growing holistic interest in the future of life on earth that has led to proposals such as One Health and planetary health ( Biehl, 2021 ), which interweave in a single effort the understanding and care of the environment, nature and human beings, in addition to growing debates in the historiography on the subject. Its editors were Regina Horta Duarte, professor at the Federal University of Minas Gerais (Universidade Federal de Minas Gerais), Gabriel Lopes, from the House of Oswaldo Cruz (Casa de Oswaldo Cruz), Natascha Stefania Carvalho De Ostos, from the René Rachou Institute or Fiocruz Minas (Instituto René Rachou/Fiocruz Minas), and Nelson Aprobato Filho, from the University of São Paulo (Universidade de São Paulo).

No less important was the first editor’s letter of volume 29 in early 2022, which reproduced a collective editorial of the forum of editors of scholarly journals of the National History Association (Associação Nacional de História, Anpuh) entitled “Support for policies that recognize the importance of academic journals in the field of History.” It was aimed at highlighting the role of scientific history journals in the circulation of knowledge, to claim the validity of evidence collected in archives and the legitimacy of historians’ interpretations, and to call attention to the importance of sustaining the editorial teams that produce periodicals (not only editors but also copyeditors, translators, secretaries, visual programmers, journalists, among others).

Also of great impact was the dossier “Material culture of Ibero-American science: museums, gardens and scientific cabinets,” organized by researchers Carolina Valenzuela, from the University of Chile (Universidad de Chile), and María Gabriela Mayoni, from the University of Buenos Aires (Universidad de Buenos Aires), which appeared in last year’s issue number 3. The valuable texts published are improved versions presented at the symposium Visual, Material and Sensory Cultures of Science, which was part of the important ninth conference of the European Society for the History of Science, held between late August and early September 2020 in Bologna, Italy. By publishing it, we also wanted to reiterate the journal’s commitment to recording what is happening at the frontiers of knowledge that is usually made public in academic events.

Before being called to hold the position of minister of Health, we had the privilege of publishing, in issue number 4, a letter from Nisia Trindade Lima in which she celebrated the 21st anniversary of the Graduate Program in History of Science and Health at the Casa de Oswaldo Cruz. In her text she argues that historical research is like a window to glimpse the future.

Finally, I would like to highlight the special issue “Transcultural histories of psychotherapies: new narratives,” organized by Professors Sonu Shamdasani and Cristiana Facchinetti. This 2022 supplement is important for two reasons. First, because it presents the complexities of the circulation and exchange of mental health knowledge and practices in Argentina, Brazil, the USA, Italy, Japan and Switzerland. Second, because it is the expression of a productive collaborative agreement dating back to 2016 between the University College London, where Sonu Shamdasani is a professor, and Casa de Oswaldo Cruz, where Cristiana Facchinetti is a researcher.

All of the above has been possible thanks to the editorial team that returned to work at the Manguinhos campus in a face-to-face or semi-presential manner (Mônica Auler, Roberta Cerqueira, Camilo Papi, Mônica Cruz Caminha, Vinícius Renaud, Marciel Mendonça, Miriam Junghans and Fernando Vasconcelos). They, and the undersigned, interacted with the scientific journalists Marina Leme and Vivian Mannheimer, who are also part of our team. Their work was fundamental for the repercussion of the journal’s texts because they spread the launching of the publication, fed the blog that is becoming a reference for other researchers who cite it, published sound bites news that attracted the readers’ attention and linked events and well-known people to the journal’s articles. Thanks to their work, and of course to the readers, the journal’s national blog had more than 460 thousand visits during 2022 (in 2021 it had 389,797). And as of May 2023, we had more than 10,500 followers on the national Facebook page (published in Portuguese). As for Facebook, the international account (with news in Spanish and English) currently has 5,200 followers, a significant increase from 4,900 in 2021. On Twitter – where we publish content in Portuguese, English and Spanish – we currently have 4,255 followers, an increase of almost 500 followers compared to the beginning of 2022. An important feature of this social media is that, unlike previous years when the journal’s audience was basically made up of readers from Latin American countries, now the majority (30%) is concentrated in the United States, followed by Brazil (15%) and the rest of the percentage is shared by readers from Mexico, Argentina, Peru, Portugal, Spain and other countries. In this way, the blog makes a decisive contribution to the internationalization of the journal, an important achievement 10 years after its creation.

This internationalization is beginning to be seen more slowly in the submissions received. In 2022, 172 texts were submitted and processed for all sections of the journal, 67% of which came from Brazilian authors. The countries with the next highest number of submissions were Portugal, Argentina, Colombia and Chile. It should also be noted that, in 2022, the United States, United Kingdom, Spain, Uruguay and Peru each submitted between two and five articles. In global terms, this did not mean a decrease with respect to 2021 because in that year 165 manuscripts were received.

To conclude, I will write what I think about the immediate future. In the first months of 2023 we implemented continuous publication system. This system consists of the electronic publication of the journal articles, individually or in small groups, as they are approved and processed internally (i.e. after a careful linguistic revision, standardization of references, tables and images, and other tasks of the proofreaders). This replaces the publication of articles by number, being valid from now only the volume indicating the year. The advantages of the continuous flow will allow the faster dissemination of our authors’ research and increase the visibility of the journal. We also foresee renewed commitments to open science, new instructions to authors and the end of the section “Testimonials from Covid-19” – which does not mean that we will no longer receive papers on the subject for all sections. It is good to emphasize that we agree with the recommendations of the health authorities, especially in relation to vaccination, who warn that a resurgence of an ailment that still exists would not be surprising.

We are very grateful to all the authors, reviewers, section editors and members of the editorial staff who allowed our journal to have a special visibility and prestige in Brazil and much of the world. Finally, our special recognition goes to Marcos José de Araújo Pinheiro, director of Casa de Oswaldo Cruz, the Fiocruz unit where we work, for his great support to the journal.
